# Prediction of global marginal land resources for *Pistacia chinensis* Bunge by a machine learning method

**DOI:** 10.1038/s41598-022-09830-5

**Published:** 2022-04-07

**Authors:** Shuai Chen, Mengmeng Hao, Yushu Qian, Fangyu Ding, Xiaolan Xie, Tian Ma

**Affiliations:** 1 Institute of Geographic Sciences and Natural Resources Research, Chinese Academy of Sciences, 11A Datun Road, Chaoyang District, Beijing, 100101 China; 2 College of Resources and Environment, University of Chinese Academy of Sciences, Beijing, 100049 China

**Keywords:** Climate-change mitigation, Biofuels

## Abstract

Biofuel has attracted worldwide attention due to its potential to combat climate change and meet emission reduction targets. *Pistacia chinensis* Bunge (*P. chinensis*) is a prospective plant for producing biodiesel. Estimating the global potential marginal land resources for cultivating this species would be conducive to exploiting bioenergy yielded from it. In this study, we applied a machine learning method, boosted regression tree, to estimate the suitable marginal land for growing *P. chinensis* worldwide. The result indicated that most of the qualified marginal land is found in Southern Africa, the southern part of North America, the western part of South America, Southeast Asia, Southern Europe, and eastern and southwest coasts of Oceania, for a grand total of 1311.85 million hectares. Besides, we evaluated the relative importance of the environmental variables, revealing the major environmental factors that determine the suitability for growing *P. chinensis*, which include mean annual water vapor pressure, mean annual temperature, mean solar radiation, and annual cumulative precipitation. The potential global distribution of *P. chinensis* could provide a valuable basis to guide the formulation of *P. chinensis*-based biodiesel policies.

## Introduction

At the COP 26 UN Climate Change Conference hosted by the United Kingdom partnering with Italy, one of the major goals set out to put climate change under control is to secure global net-zero emissions by mid-century and keep 1.5 degrees within reach^1^. As an important intervention to combat climate change and meet emission reduction targets, the development of biofuel– mostly ethanol and biodiesel– has greatly attracted worldwide attention due to the growing demands for cleaner energy sources^2,3^. Modern bioenergy provided 5.1% of total global final energy demand in 2019 when global biofuel production increased 5% this year^4^. In 2020, however, global biofuel production fell 5% owing to the influence of the COVID-19 pandemic on overall energy demand in transportation, of which ethanol production went down 8%, and biodiesel production remained stagnant^4^. Due to the wide range of biodiesel feedstocks, its production is found on a broader scale than that of ethanol^5^. For instance, the global biodiesel output mainly comprises production from 11 countries, while most of the ethanol yield comes from merely 2 countries. Hence biodiesel is considered a very prospective renewable energy.

*Pistacia chinensis* Bunge (*P. chinensis*) is one of the promising species for producing biodiesel given some of its outstanding characteristics, including drought resistance, tolerance to cold climate, poor, acid or alkaline soils^6,7^. Meanwhile, the *P. chinensis*-based biodiesel also has some comprehensive advantages in oil yield and its conversion rate, as well as biodiesel quality^7^. Another strength of this biodiesel is that it is mainly composed of fatty acids with carbon chain length 16–18, which is quite close to the main component of the fossil diesel (C15–C19)^8^. Currently, research of *P. chinensis* focuses on its production technology^9–12^, environmental effects^13^, and economic benefits^14^, etc. Although marginal land resource assessment has been considered the underpinning for large-scale exploitation of *P. chinensis*-based biodiesel, there is only limited research conducted on this topic so far and only restricted to plants in China. Lu et al. evaluated the marginal land references suitable for developing *P. chinensis*-based biodiesel in China with multiple datasets (natural habitat, remote sensing-derived land use, meteorological and soil data) and geoinformatics techniques^15^. Wang et al. used the same methodology to obtain the marginal land and assessed potential productivity of *P. chinensis*-based biodiesel as well as other woody energy crops in China^16^. A more finely conducted research by Yin et al. comprehensively evaluated the marginal land resources suitable for the cultivation *P. chinensis* in Shaanxi province, China, with the multi-factor integrated assessment^17^. The most extensive research on the potential spatial distribution of *P. chinensis* at present is to simulate the distribution in Asia. Fu et al. used the multi-factor analysis to identify marginal land in Asia with multiple associated datasets and characteristics of *P. chinensis*^18^. To sum up, we found that the multi-factor integrated analysis was currently used to extract the marginal land suitable for *P. chinensis*. However, there are several main problems remaining unsolved in current studies: (1) results from the multi-factor integrated analysis and the corresponding data used to determine the marginal land are relatively coarse; (2) the relative influences of environmental factors on the marginal land distribution have not been evaluated; (3) the distribution of global potential marginal land for *P. chinensis*-based biodiesel has not been estimated.

Compared to the multi-factor analysis method, the machine learning method could identify the environmental niche of *P. chinensis* and provide the relative importance of influencing factors^19^. Therefore, we use a machine learning method, boosted regression trees (BRT), which has been successfully used in cassava and silvergrass^20^ to simulate the worldwide potential distribution of *P. chinensis* with environmental factors and *P. chinensis* occurrence records from the perspective of environmental suitability.

## Methods

The study is carried out through the following steps: firstly, we applied BRT models combined with the presence records and a set of environmental predictors to predict the environmental suitability for *P. chinensis*; secondly, we identified the major environmental factors that determine the suitability for P. chinensis by evaluating the relative contribution rate of each environmental variable; thirdly, we overlayed the land-use map with the suitable regions obtained from the first step to extract the marginal land suitable for growing *P. chinensis*, and analyzed the land-use composition of qualified marginal land resources.

We acquired the geographical distribution data of *P. chinensis* from the Global Biodiversity Information Facility (GBIF, http://www.gbif-uat.org/), which contains 1923 georeferenced records. These records were aggregated up to grid cells with a common spatial resolution of 0.05°, and 670 samples were obtained as presence records. The same number of points were selected as pseudo-absence records based on the broad environmental suitability of *P. chinensis*. Specifically, the pseudo-absence records were randomly selected within areas where the mean annual temperature is either lower than 5.8 °C or beyond 28.4 °C, and where the annual mean precipitation is less than 400 mm or more than 1900 mm, these areas have previously been shown unsuitable for *P. chinensis* survive^15^.

The set of environmental predictors consists of 3 types: climate, soil, and topography, which are closely associated with the distribution of *P. chinensis*. We included mean annual water vapor pressure, mean annual temperature, mean solar radiation, and annual cumulative precipitation to reflect the climatic conditions for *P. chinensis*. These data were derived from the WorldClim version 2.0 database (http://www.worldclim.com/). Soil water content, soil class, and soil depth were obtained from the World Soil Information (http://www.isric.org/) to mirror the soil characteristics. The elevation and slope data from the website of the Consultative Group on International Agricultural Research (CGIAR) Consortium for Spatial Information (http://srtm.csi.cgiar.org) were used to simulate the topographical conditions. Besides, land-use data obtained from NASA's Earth Observatory Group (https://lpdaac.usgs.gov/) were used to distinguish marginal lands. All above raster data were resampled to the same coordinate system with a spatial resolution of 0.05° using ArcGIS 10.2 software^21^.

Boosted regression tree (BRT) models were applied to quantify the relationship between environmental predictors and occurrence records of *P. chinensis* as well as predict its potential distribution. As an advanced machine learning technique, BRTs have been extensively used in ecological studies. BRTs use regression trees and gradient boosting to iteratively fit and combine multiple regression tree models to improve stability and predictive accuracy^22^. The virtue of BRTs is their ability to model complex ecological systems for (1) neatly accommodating continuous and categorical predictors, missing data, and outliers without requiring data pre-transformation, and (2) calculating complex nonlinear relationships between the variables^23^.

All BRTs were fitted in R 3.3.3^24^, using the ‘dismo’ and ‘gbm’ library^25,26^. We ran the performed 30 bootstrapped BRT models with a random sample of 1340 grid cells and averaged the results from simulations to ensure reliability. In addition, we used the area under the curve (AUC) of the receiver operating characteristic (ROC) curve to evaluate the performance of BRT models^27,28^. We also applied ten-fold cross-validation procedures to select the optimal number of regression trees for each model using the “gbm.step” function and to avoid overfitting. We used the relative contribution (RC) percentage to evaluate the relevance of each predictor in BRT models and generated partial dependence plots to describe the effect of each predictor on the potential distribution of *P. chinensis*, while other independent variables were taken as mean or constant. Each cross-validation BRT model built with training data (50%) yields a cross-validated AUC, while its model predictions with testing data (50%) provide the estimation of a testing AUC.

## Results

### Accuracy of global suitability prediction for *P. chinensis*

**Figure 1:**
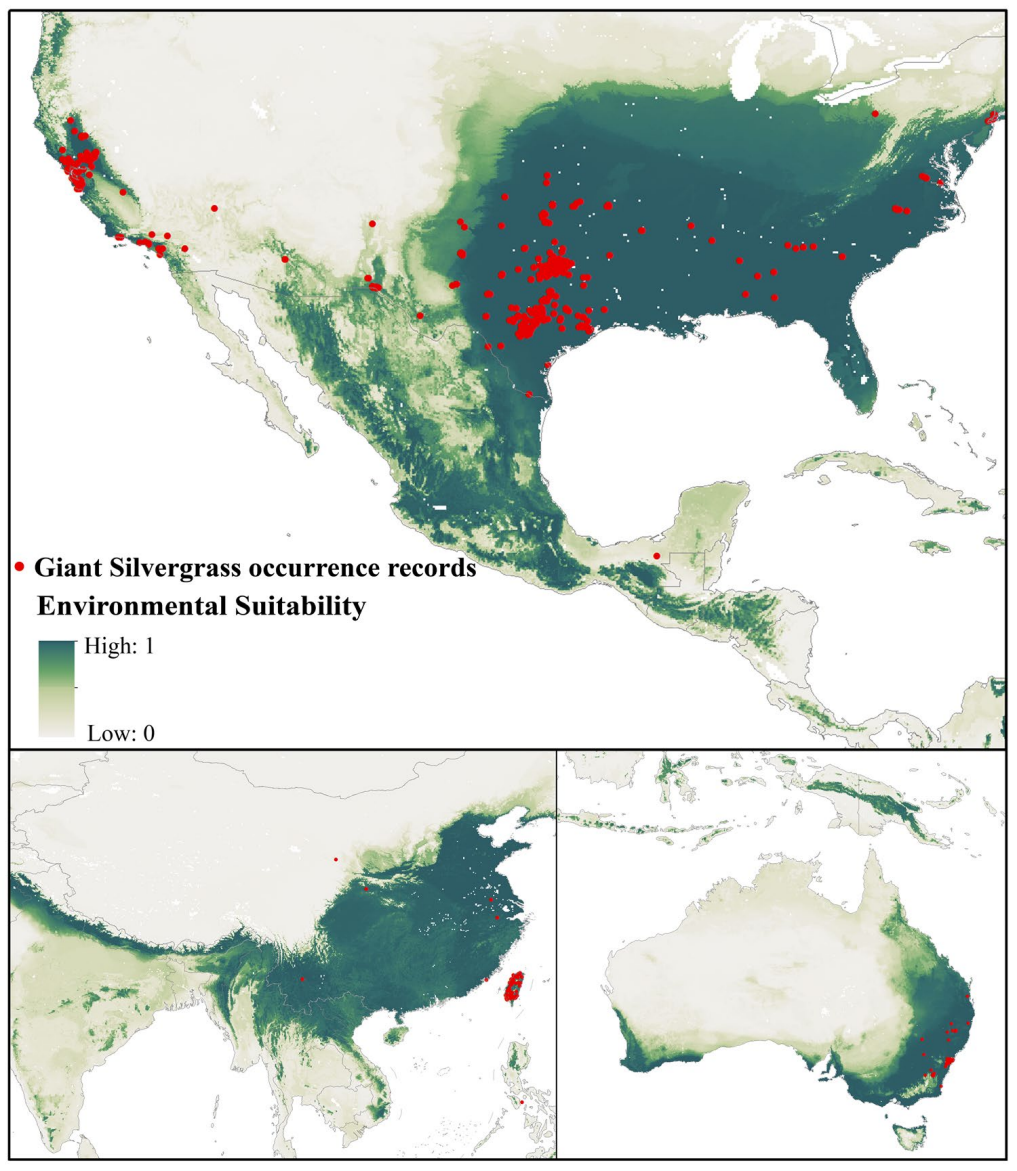
Distribution of global *P. chinensis* occurrence records layering over the global environmental suitability for *P. chinensis* to visually verify the accuracy of model prediction [Figure was created with ArcGIS Desktop (ESRI, Inc, Version 10.2, https://desktop.arcgis.com)].

Layering the global occurrence points of our plant on its environmental suitability map (Fig. 1), we find the consistency that these points of existing plants mostly appear in regions of high suitability, which could indicate the good performance of BRT model.

To further evaluate the accuracy of our simulation, we calculated the statistic index under tenfold cross-validation procedure, obtaining the training data AUC = 0.995 ± 0.001 and validation data AUC = 0.995 ± 0.003, which could give credibility to the outputs of the BRT model.

**Figure 2:**
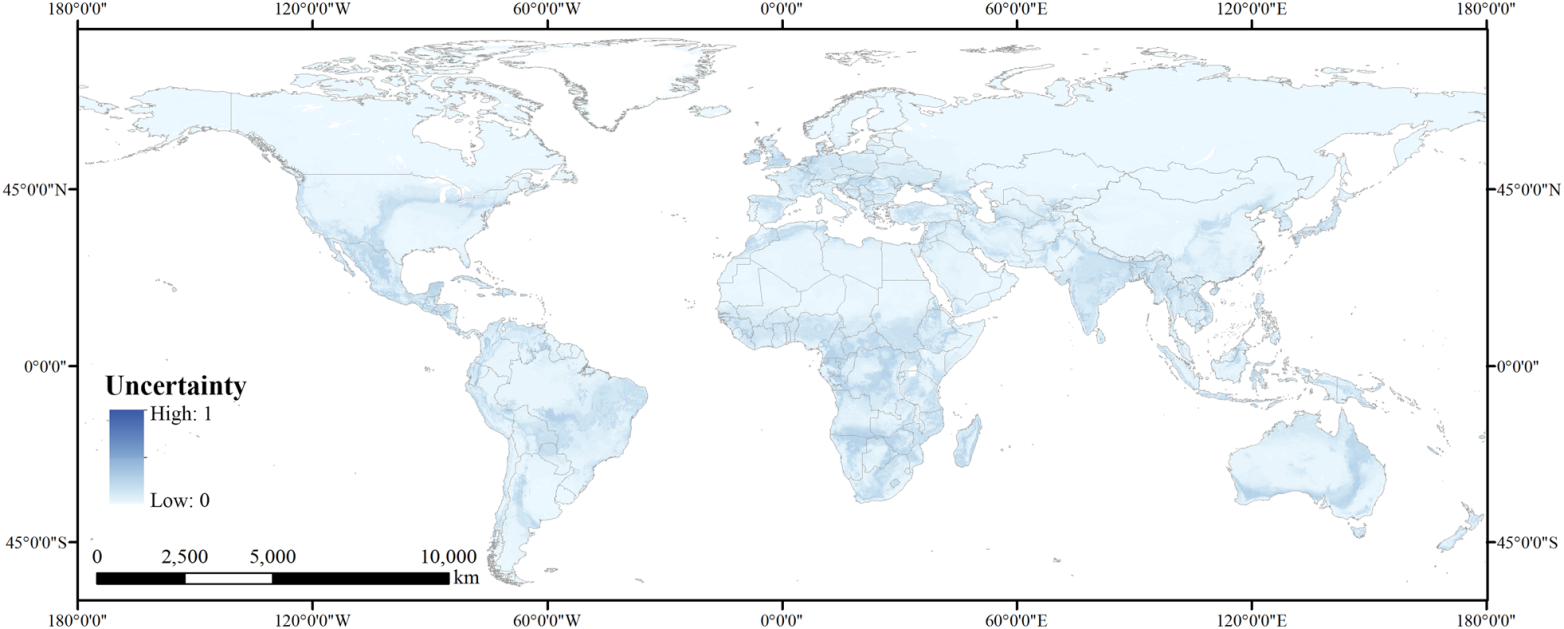
Visualized uncertainty in spatial prediction on the basis of standard deviation values computed for each pixel across the model ensemble. [Figure was created with ArcGIS Desktop (ESRI, Inc, Version 10.2, https://desktop.arcgis.com)].

In addition, we used the standard deviation values to quantify the uncertainty of the spatial prediction. The visualized quantification in Fig. 2 presented relatively low uncertainty, which could further validate the results of model simulation.

### Relative contribution of the environmental covariates

Given that the accuracy of our model prediction has been validated through multiple means, the reliability of variables’ relative contribution in the simulating process were therefore guaranteed. Table 1 shows the relative contribution of the environmental covariates related to predicting the global distribution of *P. chinensis*, concurring with our expectation that climate variables substantially influence identifying global land resources for growing *P. chinensis*. The top four variables determining the suitability for *P. chinensis* all fall into the category of representing climate conditions, which are mean annual water vapor (58.76% [95%CI 57.54–59.98]), mean annual temperature (24.33% [95%CI 23.08–25.58]), mean solar radiation (5.49% [95%CI 4.67–6.31]), annual cumulative precipitation (5.26% [95%CI 4.17–6.35]), accounting for totally 93.84% of the contribution. By comparison, soil and topographical factors exhibit far less importance: soil water content (3.90% [95%CI 3.26–4.53]), soil class (0.41% [95%CI 0.28–0.53]), soil depth (0.24% [95%CI 0.28–0.53]), elevation (1.34% [95%CI 1.14–1.54]), slope (0.27% [95%CI 0.22–0.33]).

**Table 1:** The relative contribution of each spatial predictor variables.

Environmental conditions	Covariates	Mean (%)	95% CI
Climate conditions	Mean annual water vapor pressure	58.76	57.54–59.98
	Mean annual temperature	24.33	23.08–25.58
	Mean solar radiation	5.49	4.67–6.31
	Annual cumulative precipitation	5.26	4.17–6.35
Soil conditions	Soil water content	3.90	3.26–4.53
	Soil class	0.41	0.28–0.53
	Soil depth	0.24	0.16–0.32
Topography condition	Elevation	1.34	1.14–1.54
	Slope	0.27	0.22–0.33

### Potential marginal land resources suitable for *P. chinensis*

#### Global distribution of potential marginal land resources for *P. chinensis*

A necessary step prior to estimating the worldwide potential marginal land available for proper cultivation of *P. chinensis* is evaluating the global suitability for growing this plant.

**Figure 3:**
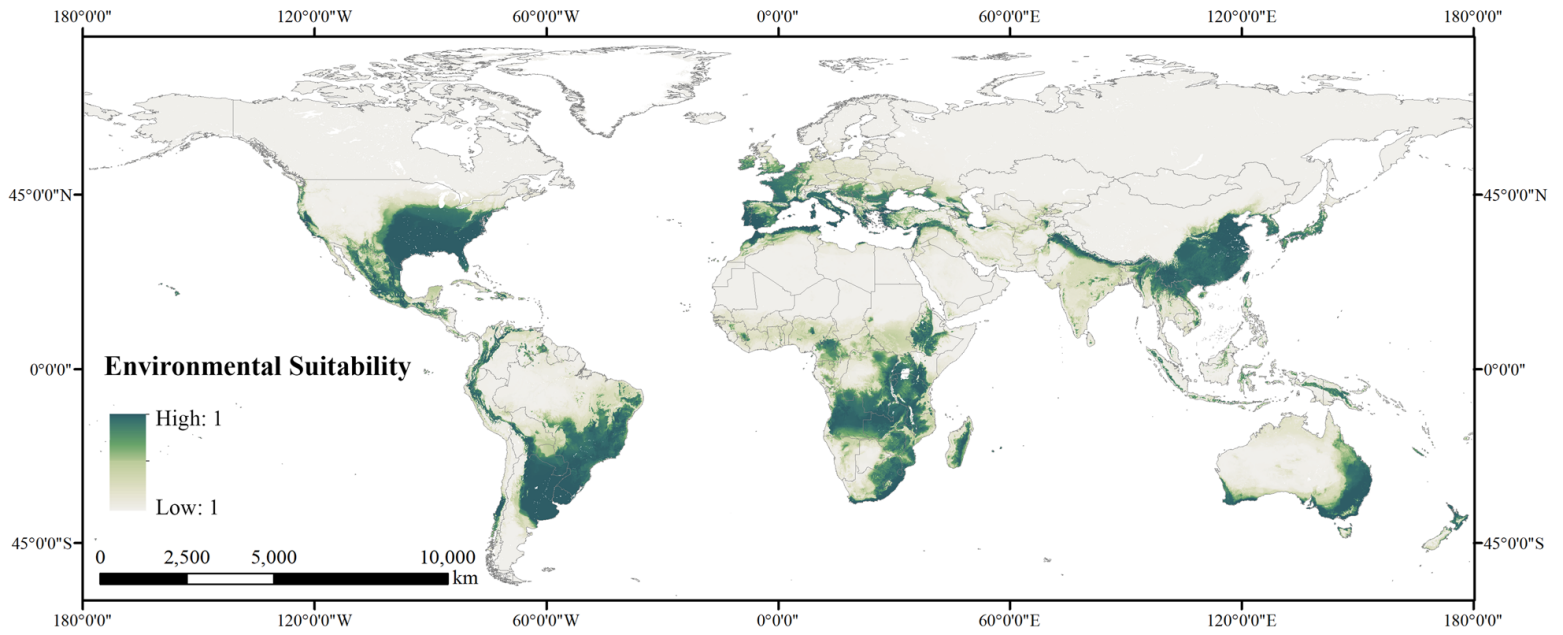
Predicted global environmental suitability for *P. chinensis* ranging from high (dark greenish blue) to low (light gray) [Figure was created with ArcGIS Desktop (ESRI, Inc, Version 10.2, https://desktop.arcgis.com)].

In the global map (Fig. 3), we distinguished the environmentally suitable regions for growing *P. chinensis*, covering a broad span of latitudes from 45 degrees north and 45 degrees south. Generally, the map exhibits a descending trend in suitability from coasts toward inland, excluding a few suitable regions in Africa and areas along borders between countries.

**Figure 4:**
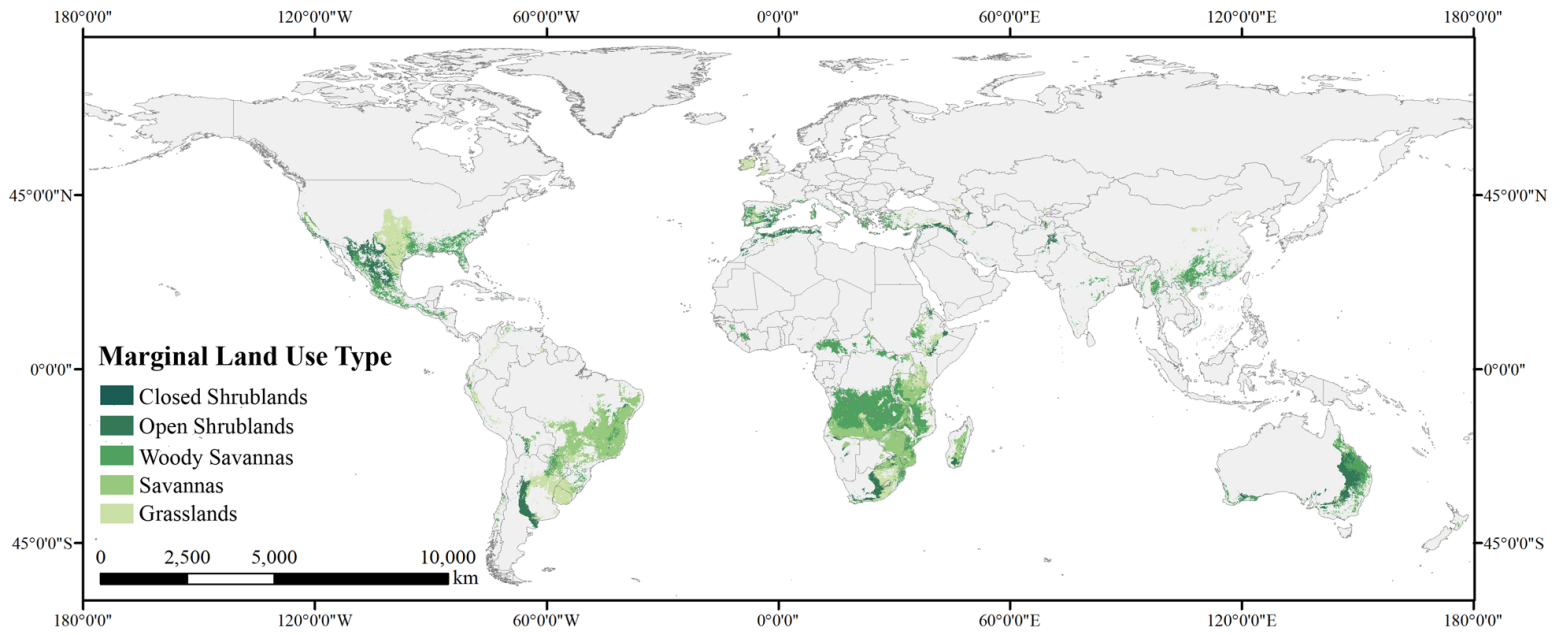
Map of the potential marginal land resources suitable for *P. chinensis* planting classified by land-use type [Figure was created with ArcGIS Desktop (ESRI, Inc, Version 10.2, https://desktop.arcgis.com)].

To identify potential land resources for planting, we determined whether a 5 × 5 km^2^ grid cell is suitable for *P. chinensis* by setting 0.5 as the value of the threshold. After that, when distinguishing potential marginal land out of all land resources for growing *P. chinensis*, it is our understanding that in case of compromising environment preservation and productivity, we should include only a few land-use types: savannas, shrublands, and grasslands. The resulting map is presented in Fig. 4.

The quantified results show that globally, there are 1311.85 million hectares of marginal land in total for proper *P. chinensis* cultivation, mostly distributed in Southern Africa, the southern part of North America, the western part of South America, Southeast Asia, Southern Europe, and eastern and southwest coasts of Oceania.

Africa, though no *P. chinensis* has been found there yet, has the greatest amount of land resources for the planting, which is 528.19 million hectares in total, for growing this plant. According to the map, the majority of Angola, Zambia, Mozambique, Tanzania, Zimbabwe, and Malawi, and a considerable proportion of Zaire, South Africa, Ethiopia, Madagascar, and Kenya has potential for future cultivation. South America has the next largest amount of marginal land to Africa. The total 304.44 million hectares are mainly distributed in West Brazil, central Argentina, Uruguay, Paraguay. We also find a relatively small portion of land resources in Peru, Bolivia, Chile, Ecuador, Venezuela, and Colombia. North America possesses 222.99 million hectares of plantable marginal land for our plant, mainly found in the United States and Mexico. Asia, where *P. chinensis* is native to, only owns third to last marginal land for growing it. The majority of the total 106.18 million hectare land resources are from countries in Southeast Asia, including China, Myanmar, and Vietnam. Also, part of Turkey, India, Iran, Iraq, and the bordering region between Pakistan and Afghanistan are also found suitable for potential cultivation. In Oceania, marginal land resources are mostly distributed in coastal regions. The total amount of land resources is 101.04 million hectares, of which 97.99% are found in Australia. In Europe, we find the least land resources among all continents, which are mainly distributed in Spain, Portugal, Ireland, the United Kingdom, Greece, Italy, and France, amounting to 49.01 million hectares.

#### Global land-use composition of potential land resources for *P. chinensis*

Classification of the global marginal land resources by land-use type could reveal the land-use structure of available land for *P. chinensis* all over the globe, indicating that the land resources mostly consist of woody savannas, savannas, grasslands, and open shrublands, leaving only a tiny fraction of marginal land as closed shrublands.

Our calculation shows that woody savannas take up the largest proportion of land resources, accounting for 37.72% (494.80 million hectares) of the total. In addition, savannas (358.71 million hectares), grasslands (257.10 million hectares), and open shrublands (198.65 million hectares) share fair proportions of all the plantable land resources, which are 27.34%, 19.60%, and 15.14%, respectively. In comparison, close shrublands (2.59 million hectares) make an almost negligible contribution to the marginal land for planting, sharing 0.20% of the total.

**Figure 5:**
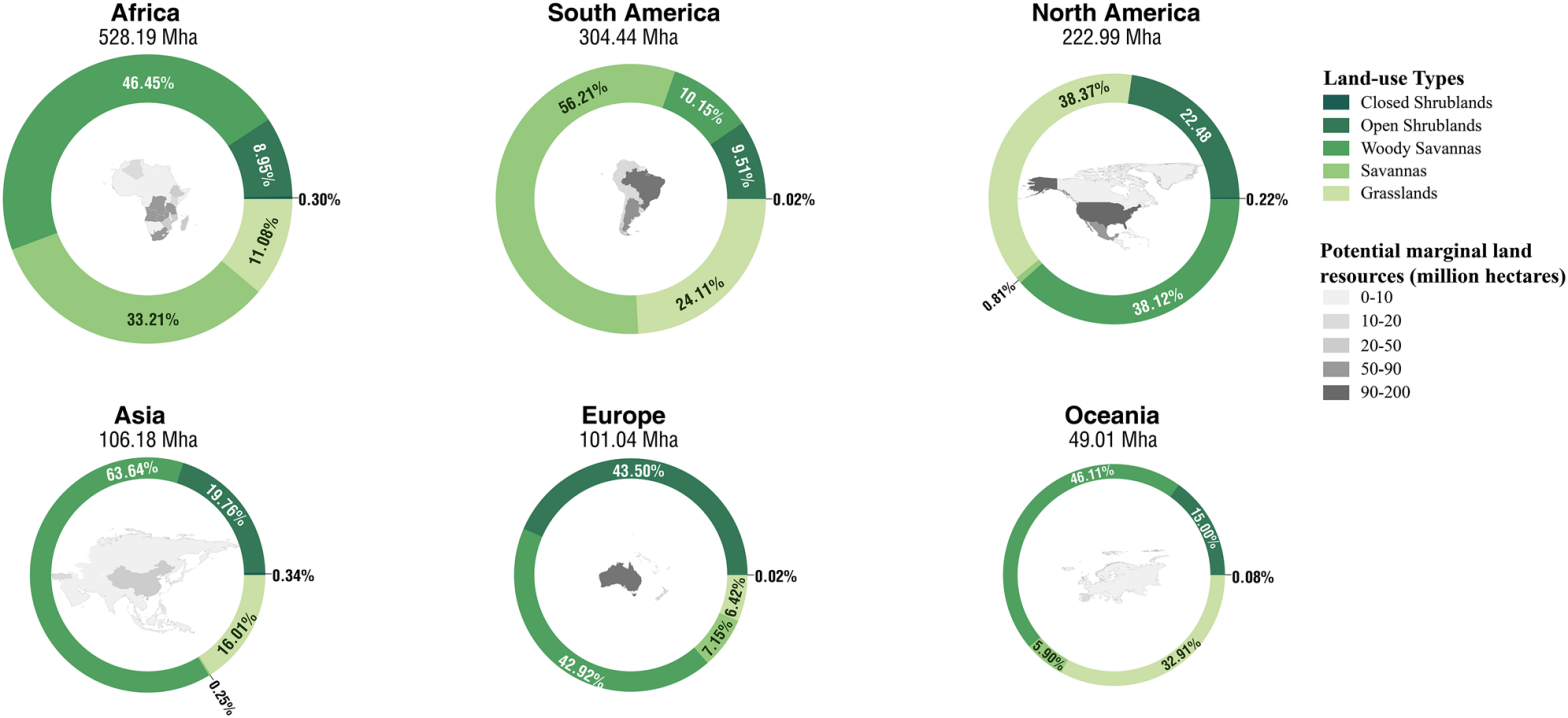
Pie charts depicting the size (thickness of the circles) and land-use composition (illustrated by different greens on the circles, representing different types of land use) of available marginal land in each continent for planting *P. chinensis* [Figure was created with Microsoft Excel (Microsoft, Version 16.0, https://www.microsoft.com/en-us/microsoft-365/excel) and Adobe Illustrator (Adobe Inc, Version 25.0, URL: https://www.adobe.com/products/illustrator.html)].

Moreover, we managed to calculate the percentage of all types of land use for planting *P. chinensis* in each continent, respectively, presented in the pie charts made below (Fig. 5). It is not surprising that we find woody savannas have the leading position in marginal land resources of several continents, including Africa, Asia, Europe, and Oceania. The following type is savannas, comprising the largest proportion of marginal land in South America.

Furthermore, quantification results for countries with more than 10 million hectares of land resources for *P. chinensis* cultivation in all continents are itemized in descending order by size in Table 2. The table shows that 9 of the total 21 countries in the list are from Africa, amounting to 443.95 million hectares, accounting for 33.84% of the total land resources. However, the country with the largest amount of available marginal land is Brazil (190.58 million hectares) from South America, which is mainly comprised of savannas. In terms of the land-use type in the leading position of these countries, 7 of the 21 countries are primarily distributed with woody savannas (236.83 million hectares in total), while 6 of them are mainly distributed with grasslands (156.90 million hectares), followed by 5 countries with savannas (235.73 million hectares), 3 countries with open shrublands (87.58 million hectares). The results on the national scale are generally consistent with that on the continental scale, indicating the substantial share of countries in Africa as well as woody savannas in total available marginal land.

**Table 2:**
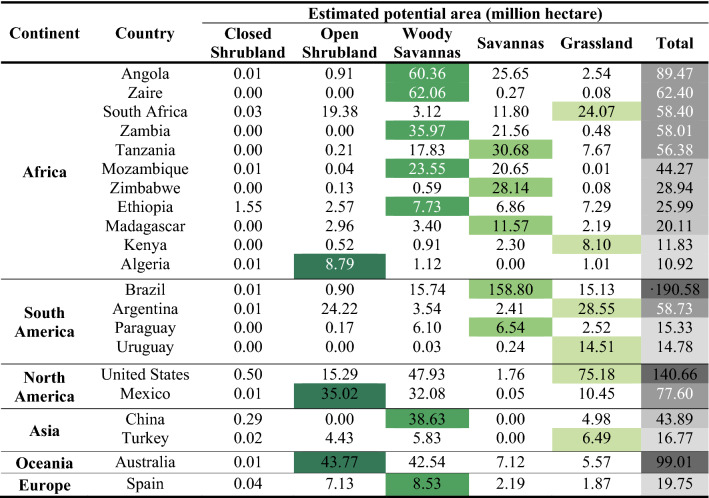
Potential marginal land resources suitable for *P. chinensis* in major global regions and the countries with the area of suitable land > 10 million hectares.

The cells with the total amounts of each country’s land resource are marked with different grays varying from dark to light by the size of available land, while cells with the amounts of the largest land resource type of each country are marked with different greens varying by the land-use type.

## Discussion

In this study, an advanced machine learning algorithm, boosted regression trees, is used to obtain the potential spatial distribution of *P. chinensis*, integrated with consideration of crop growing conditions and marginal land types. Although the occurrence records for *P. chinensis* are mainly located in China, the United States, and Australia, the potential marginal land suitable for *P. chinensis* is abundant in many other states, amounting to a total of 1311.85 million hectares. Fu et al. use the multiple factor analysis method to obtain the marginal land suitable for *P. chinensis* in their study, where the number of land resources ranged from 26.5 million hectares to 214 million hectares in Asia, respectively. In our study, 106.18 million hectares of marginal land suitable for *P. chinensis* is identified, which is reasonable compared with the research of Fu et al. on China. Wang et al. calculated that marginal land suitable for *P. chinensis* is 22.28 million hectares. Assessment of Lu et al. showed that the 19.90 million hectares of marginal land suitable were found for *P. chinensis* in China^15^. In this study, we find 43.89 million hectares of marginal land suitable for *P. chinensis* in China, which is larger than other research. This discrepancy is mainly because of the differences in constraints between the two studies. Lu et al. considered mostly the national policy, including the ecological red line and natural reserve that cannot be exploited. Besides, the mean cultivation index of 60% is also considered in their study. Overall, the results in this study, by comparison with other research, are scientific and credible.

The potential spatial distribution of *P. chinensis* obtained in this study refers to the macroscopic distribution from the perspective of environmental suitability. With the result of total cultivatable marginal land in this study, fruit oil content and yields per hectare, biodiesel conversion rate we found from previous literature, we managed to roughly calculate the global biodiesel produced from *P. chinensis*, which ranges from 375.54 to 667.31 billion liters assuming that 30% of the marginal land resources can be used for *P. chinensis* cultivation^7,29–31^. Given the GHG (greenhouse gas) abatement offered per liter of biodiesel used in lieu of gasoline or diesel, using *P. chinensis*-based biodiesel could reduce 0.27 to 1.95 gigaton CO_2_e emissions^32^, meeting 0.98% to 6.96% of the annual reduced emissions goal (28 GtCO2e) to limit global warming to 1.5 °C. More optimistic estimation is to use 50% of available marginal land to grow *P. chinensis*, the obtained 625.90 to 1112.19 billion liters of *P. chinensis*-based biodiesel used to replace gasoline or diesel could reduce 0.46 to 3.25 gigaton CO_2_e emissions, meeting 1.63% to 11.60% of the annual reduced emissions goal.

However, when zooming in and focusing on a particular area, specific local policies should be considered, which include ecological protection, economic and environmental benefits, etc., to ensure the sustainable development of bioenergy in each region^33–35^. For example, despite the results in this study suggesting that Africa has the most marginal land resources available for energy development by far, it is not suitable for developing *P. chinensis* based biodiesel on a large scale because its current condition fails to meet the criteria of modern bioenergy technologies^36^. Furthermore, the development of *P. chinensis* should also balance food, fodder, and fuel supply in land use planning in Africa^37^. In addition, for the economic and environmental benefits, the life cycle assessment of *P. chinensis*-based biodiesel should be considered to determine the development area of marginal land^38,39^.

The limited occurrence records are the major limitation of this study. The global potential spatial distribution of marginal land suitable for *P. chinensis* would be more precise if more samples were recorded and available for download.

## Conclusion

As one of the prospective energy plants, estimating the potential marginal land resources for *P. chinensis* cultivation would be conducive to exploiting bioenergy yielded from this species. Boosted regression tree (BRT), the machine learning method we applied in this study, enabled us to evaluate the relative importance of the environmental variables, revealing the primary influence on defining the qualification for growing *P. chinensis* of several indicators that represent climate conditions, including mean annual water vapor pressure, mean annual temperature, mean solar radiation, and annual cumulative precipitation.

Moreover, aided by this model, we map the suitability for growing *P. chinensis* across the planet, based on which we derive the marginal land resources distributed in each continent. Most of the qualified marginal land is found in Southern Africa, the southern part of North America, the western part of South America, Southeast Asia, Southern Europe, and eastern and southwest coasts of Oceania, for a grand total of 1311.85 million hectares.

## Data Availability

The data sources that support the findings of this study can be found in the Methods section.
